# Potential of siphonaxanthin, a green algal carotenoid, to prevent obesity and related diseases

**DOI:** 10.1007/s11418-025-01897-4

**Published:** 2025-04-12

**Authors:** Yuki Manabe, Tatsuya Sugawara

**Affiliations:** Division of Applied Biosciences, Graduate School of Agriculture, Kyoto University, Kitashirakawa Oiwake-cho, Sakyo-ku, Kyoto, 606-8502 Japan

**Keywords:** Carotenoid, Obesity, Fatty liver, 3T3-L1 preadipocyte, KK-Ay mouse, Ob/Ob mouse

## Abstract

The increasing prevalence of obesity and its related diseases, including diabetes mellitus and metabolic dysfunction-associated fatty liver disease, has become a significant social problem. These diseases are believed to be preventable through healthy diet and exercise habits, and the investigation of food ingredients that are useful for prevention of these diseases is actively ongoing. Carotenoids are the major lipophilic pigments responsible for yellow-to-red colors in our diet, and the ingestion of certain carotenoids has been reported to prevent obesity. For example, β-carotene suppresses adipogenic differentiation of mouse preadipocyte line 3T3-L1 through its provitamin A activity. Fucoxanthin, a carotenoid found in brown algae, also has the similar effect via a different mechanism and is used as an active ingredient in foods with functional claims in Japan. In contrast, siphonaxanthin, a carotenoid found in some green algae such as *Caulerpa lentillifera* (commonly known as sea grape), exhibited stronger biological activities than other carotenoids in cell-based studies; it significantly suppressed adipogenic differentiation of 3T3-L1 cells even at low concentrations where β-carotene and fucoxanthin did not show inhibitory effects. However, its practical applications have not yet been realized. This review summarizes the studies on the anti-obesity effects of carotenoids and discusses the potential of siphonaxanthin as a novel functional food ingredient.

**Graphical Abstract:**

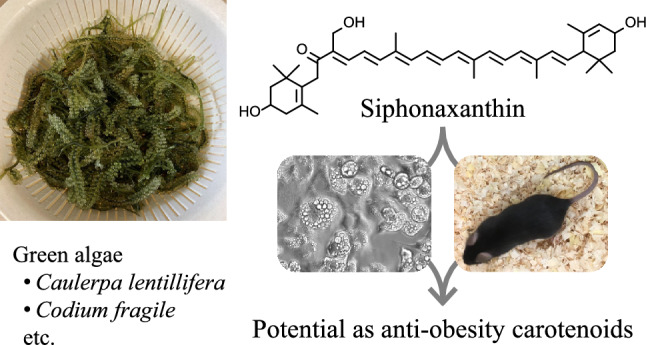

## Introduction

According to the Ministry of Health, Labor, and Welfare, in 2023, approximately 30% of adult men and 20% of adult women in Japan will have a body mass index (BMI; the ratio of weight in kilograms to squared height in meters) of 25 or higher and will be considered obese. Obesity is a condition in which energy intake exceeds energy consumption and excess energy is stored as fat in the body. In particular, visceral adiposity, in which fat accumulates around the internal organs, tends to cause insulin resistance, a condition in which insulin does not function sufficiently. As a result, blood glucose levels become unregulated, and the risk of developing diabetes mellitus increases. In addition, accumulation of visceral fat increases the influx of fat into the liver via the release of fatty acids from adipocytes. The accumulation of fat in hepatocytes leads to the development of metabolic dysfunction-associated fatty liver disease (MAFLD), which may progress to metabolic dysfunction-associated steatohepatitis (MASH), cirrhosis, and eventually hepatocellular carcinoma. Approximately 25% of individuals undergoing health checkups and physical examinations have MAFLD. The number of people affected by MAFLD and obesity is substantial and has become a significant social issue. Both these diseases are believed to be preventable through healthy diet and exercise habits, and the investigation of food ingredients that are useful for prevention is also actively ongoing.

**Fig. 1:**
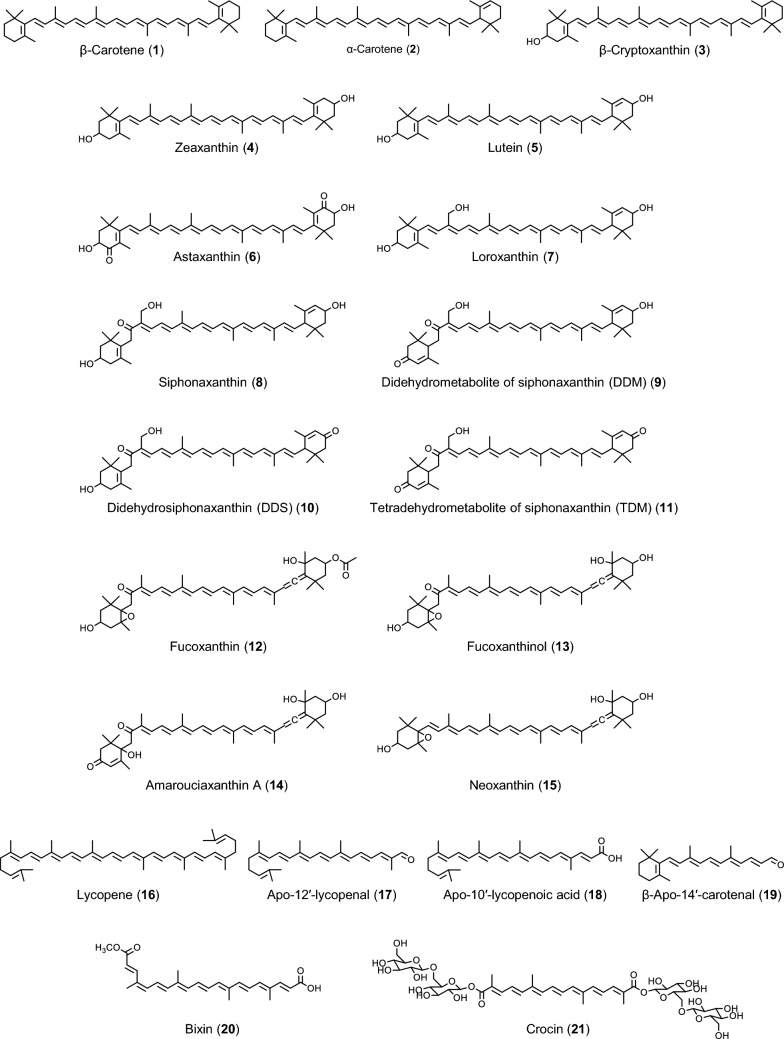
Chemical structures of carotenoids discussed in this review

Carotenoids are widely distributed natural pigments responsible for the yellow-to-red coloration. They are produced de novo by photosynthetic organisms as light-harvesting pigments and antioxidants that protect against sunlight damage. Because the amount of sunlight reaching land and underwater differs greatly, algae develop distinctive carotenoids compared to terrestrial plants. Red, brown, and green algae contain division- or class-specific carotenoids, and taxonomic studies of algae have been conducted using carotenoid composition [1]. Green algae are closely related to land plants, and several of them contain lutein (**5**) similar to land plants. Green algal species that contain little or no lutein contain its derivatives, loroxanthin (**7**) or siphonaxanthin (**8**) (Fig. 1). According to our analysis of green algae inhabiting the Amakusa and Oki islands, *Chaetomorpha moniligera*, *Microdictyon japonicum*, *Ulva pertusa*, and *Ulva compressa* contain lutein; *Blidingia minima* and *Chaetomorpha crassa* contain loroxanthin and lutein; *Codium fragile*, *Codium barbatum*, and *Caulerpa scalpelliformis* contain siphonaxanthin and lutein; and *Cladophora wrightiana*, *Codium subtubulosum*, and *Codium spongiosum* contain siphonaxanthin; and there are no species that do not have lutein, loroxanthin, or siphonaxanthin [2]. The carotenoid content differs depending on the environment and season. Compared with those collected in April from the similar environment, *Ulva pertusa* has 157 ± 52 nmol/g dry weight of lutein, *Chaetomorpha crassa* has 462 ± 17 and 90 ± 8 nmol/dry weight of loroxanthin and lutein, respectively, and *Codium spongiosum* has 150 ± 27 nmol/dry weight of siphonaxanthin [2].

Lutein (**5**) is found in the usual diet because of leafy vegetables, and its biological activity and health benefits have been extensively investigated. However, the effects of loroxanthin (**7**) and siphonaxanthin (**8**) have not been fully investigated. To the best of our knowledge, loroxanthin is not found in edible algae, but in an edible Corbicula clam, *Corbicula sandai*, an endemic species in Lake Biwa and its water system in Japan [3]. Unlike loroxanthin, siphonaxanthin is found in some edible green algae including *Codium fragile* and *Caulerpa lentillifera*. In Japan, *C. fragile* has been consumed since ancient times [4], and *C. lentillifera* is very popular and is currently cultivated. We have investigated the biological activities of siphonaxanthin and reported that in many cases, it shows stronger activity than the other carotenoids [4]. Siphonaxanthin exhibited a strong apoptosis-inducing effect in the human acute promyelocytic leukemia line HL-60 [5]. Among the 17 natural carotenoids tested, only siphonaxanthin suppressed advanced glycation end products-induced inflammatory responses [6]. In this review, we focus on the preventive effects of carotenoids on obesity and related diseases, including MAFLD, and discuss the potential of siphonaxanthin compared to other carotenoids.

## Effects on pancreatic lipase activity

Ingested triacylglycerol is not directly absorbed by the body and generally needs to be degraded into two fatty acids and one 2-monoacylglycerol by pancreatic lipase before absorption. Hence, the inhibition of pancreatic lipase leads to the inhibition of intestinal fat absorption. Orlistat, a pancreatic lipase inhibitor, is used clinically as an anti-obesity agent. Although their inhibitory activity was weaker than that of orlistat, some carotenoids inhibited pancreatic lipase activity. Matsumoto et al. reported that 50% of inhibition concentration (IC_50_) of fucoxanthin (**12**), fucoxanthinol (**13**), and orlistat against the pancreatic lipase were 0.66, 0.764, and 0.0068 µM, respectively [7]. The IC_50_ value of astaxanthin (**6**) was dependent on its steric structure and ranged from 64.0 to 98.8 µM, whereas that of orlistat was 0.117 µM in the same experimental condition [8]. In both the studies, the IC_50_ values of the carotenoids were much higher than those of orlistat. In contrast, Shamarao and Chethankumar reported that a lutein (**5**) degraded product prepared by exposure to sunlight in India strongly inhibit porcine pancreatic lipase with IC_50_ at 11.8 µg/mL and that IC_50_ of orlistat was 1.38 µg/mL in their experimental condition [9]. Given that the chemical structure of siphonaxanthin (**8**) is similar to that of lutein, the siphonaxanthin degraded products could inhibit pancreatic lipases. Moreover, the administration of crocin (**21**) (25 and 100 mg/kg body weight) decreased serum triacylglycerol levels after the simultaneous administration of olive oil emulsion in Sprague–Dawley rats [10]. Matsumoto et al. showed that the administration of 2 mg of fucoxanthin or fucoxanthinol suppressed intestinal triacylglycerol absorption in Wistar ST rats [7]. These studies suggested that carotenoids have the potential to inhibit intestinal fat absorption.

## Effects on adipogenic differentiation of 3T3-L1 cell line

The adipogenic differentiation of the mouse preadipocyte line 3T3-L1 is widely used to assess the anti-obesity potential of phytochemicals, including carotenoids. In 1990, Kawada et al. evaluated the effects of vitamins on the adipogenic differentiation of 3T3-L1 cells and reported the inhibitory effect of β-carotene (**1**) [11]. They also showed that a molecular mechanism underlying this inhibitory effect of β-carotene was the upregulation of retinoic acid receptor (RAR) [12]. β-Carotene would be an important source of retinoic acid in adipocytes. Mice deficient in β,β-carotene-15,15′-monooxygenase 1 (BCMO1), an enzyme responsible for production of retinoids from β-carotene, were more susceptible to diet-induced obesity even on a vitamin A-sufficient diet [13]. Lobo et al. reported that β-carotene, but not retinol, is metabolized to retinoic acid in 3T3-L1 adipocytes [14]. Given these reports, β-carotene and possibly the other provitamin A carotenoids may be useful in suppression of adipogenesis and thus obesity.

β-Cryptoxanthin (**3**) is another major provitamin A carotenoid found in fruits including mandarin orange, *Citrus unshiu*. Although β-cryptoxanthin is reported as a poor substrate for BCMO1 [15], it can also activate RAR without being metabolized to retinoids [16, 17]. Shirakura et al. reported that β-cryptoxanthin exhibited RAR-dependent inhibition of adipogenesis of 3T3-L1 cells [16]. Moreover, Hara et al. showed that β-cryptoxanthin activated RAR in immortalized cells derived from mouse inguinal white adipose tissues (WAT) more strongly than β-carotene (**1**) [18]. Given that the provitamin A activity of β-cryptoxanthin is considered weaker than that of β-carotene, RAR signaling in adipose tissue may be unique compared to that in other tissues. Detailed analyses at animal and human levels are required.

β-Apo-14′-carotenal (**19**), a β-carotene (**1**) degraded product, is a poorer substrate for BCMO1 than β-cryptoxanthin (**3**) [15] and a weak partial agonist for RAR [19]. As β-apo-14′-carotenal inhibited 3T3-L1 differentiation at concentrations lower than that activates RAR, Ziouzenkova et al. explored a mechanism of action other than RAR activation and showed that β-apo-14′-carotenal suppressed activation of peroxisome proliferator-activated receptor (PPAR) γ and retinoid X receptor (RXR) [19]. The expression of PPARγ is upregulated during the adipogenic differentiation, and heterodimers of PPARγ and RXR induce several adipogenesis related genes. Hence, PPARγ is considered a master regulator of adipogenesis and PPARγ suppression results in the suppression of adipogenesis. RAR activation also resulted in suppression of PPARγ expression and adipogenesis in 3T3-L1 cells [12, 16]. Astaxanthin (**6**) is reported to act as a PPARγ antagonist and suppress PPARγ agonist-induced adipogenesis of 3T3-L1 [20]. In contrast, activation of PPARγ results in the promotion of adipogenesis. Bixin (**20**), a carotenoid found in annatto (*Bixa orellana*) seeds, and apo-12′-lycopenal (**17**), a lycopene metabolite, act as PPARγ agonists and promote adipogenic differentiation of 3T3-L1 [21, 22]. It should be noted that pioglitazone, a synthetic PPARγ agonist, augments glucose uptake in patients with type 2 diabetes and is clinically used for insulin-resistant diabetes mellitus [23]. Bixin and apo-12′-lycopenal also increase insulin-induced glucose uptake in 3T3-L1 adipocytes [21, 22]; hence, they have a potential to ameliorate diabetes mellitus.

The investigation of chemical structures common to carotenoids with suppressive effects on adipogenesis has been conducted. Maeda et al. showed that adipogenic differentiation of 3T3-L1 cells was suppressed by fucoxanthin (**12**), a carotenoid found in brown algae, and more strongly by fucoxanthinol (**13**), a deacetylated form of fucoxanthin [24]. The same group evaluated the effects of 13 natural carotenoids on 3T3-L1 cells adipogenesis and observed a suppressive effect of neoxanthin (**15**) [25]. The authors pointed out that an allene bond is an important chemical structure for these activities [25]. We also evaluated the effects of 12 carotenoids including β-carotene (**1**) and fucoxanthin on the adipogenesis and found the suppressive effect of siphonaxanthin (**8**) [26]. Interestingly, although siphonaxanthin is not a provitamin A carotenoid and does not have an allene bond, it significantly suppressed adipogenic differentiation of 3T3-L1 cells even at low concentrations (5.0 μM) where β-carotene and fucoxanthin did not show inhibitory effects. Molecular mechanisms underlying the suppressive effect of siphonaxanthin would be different from those underlying the effects of β-carotene and fucoxanthin. According to our analyses, the suppression of Akt phosphorylation is a molecular mechanism by which siphonaxanthin suppresses the adipogenic differentiation of 3T3-L1 cells. Gopal et al. showed that lutein (**5**) suppressed Akt phosphorylation and thus adipogenic differentiation of 3T3-L1 cells [27]; hence, 3′-hydroxy-ε-ring, which is common to siphonaxanthin and lutein, would be an important chemical structure for the suppressive effect of Akt phosphorylation and subsequent adipogenic differentiation of 3T3-L1 cells.

In addition to metabolizing provitamin A carotenoids to retinoids, metabolizing 3-hydroxy-β-ring to 3-keto-ε-ring also affects the anti-adipogenesis activities of carotenoids. Amarouciaxanthin A (**14**) is a metabolite of fucoxanthinol (**13**) that suppresses the adipogenic differentiation of 3T3-L1 cells more strongly than fucoxanthinol [28]. Kotake-Nara et al. showed the same trend in 3′-hydroxy-ε,ε-caroten-3-one and lutein (**5**) [29]. We previously reported that, in siphonaxanthin (**8**)-fed mice, most of the absorbed siphonaxanthin was metabolized to three dehydrometabolites [30] and identified their chemical structures as 3,19-dihydroxy-7,8-dihydro-β,ε-carotene-8,3′-dione (didehydrosiphonaxanthin, DDS) (**10**), 19,3′-dihydroxy-7,8-dihydro-ε,ε-carotene-3,8-dione (didehydrometabolite of siphonaxanthin, DDM) (**9**), and 19-hydroxy-7,8-dihydro-ε,ε-carotene-3,8,3′-trione (tetradehydrometabolite of siphonaxanthin, TDM) (**11**) [31]. All of them have 3-keto-ε-ring, and hence they may show stronger anti-adipogenesis activities than siphonaxanthin in 3T3-L1 cells. We typically ingest various carotenoids with 3-hydroxy-β-ring that is metabolized to 3-keto-ε-ring [32]. To investigate the anti-adipogenesis activities of dietary carotenoids in 3T3-L1 cells, it is important to examine the activities of carotenoid metabolites.

## Effects on beige adipocyte differentiation of 3T3-L1 cell line

Adipocytes are classified into three types based on their color: white, beige, and brown. Brown adipocytes contain a number of mitochondria that express uncoupling protein 1 (UCP1). UCP1 resides in the mitochondrial inner membrane and converts proton motive force to heat, independent of ATP generation. Therefore, brown adipocytes play a pivotal role in thermogenesis and energy expenditure, and factors regulating UCP1 expression are attractive targets for obesity research. Serra et al. reported that β-carotene (**1**), α-carotene (**2**), and lutein (**5**) increased UCP1 expression in primary brown adipocytes, and the effect was related to their RAR activating abilities [33]. In contrast, white adipocytes contain large lipid droplets that store excess energy as triacylglycerols and generally do not express UCP1. As discussed above, the suppression of white adipocyte (3T3-L1) differentiation results in the suppression of obesity. Beige adipocytes have the characteristics of both brown and white adipocytes. They have a number of mitochondria that express UCP1 and are mainly present in WAT [34]. Some consider beige adipocytes as a third type of adipocyte that differs from brown and white adipocytes, whereas others believe that white adipocytes differentiate into beige adipocytes, which is often referred to as browning. Several food ingredients, including carotenoids, induce beige adipocyte differentiation of 3T3-L1 white adipocytes. Since differentiation into beige adipocytes leads to energy expenditure via UCP1, food ingredients that promote this process are expected to exhibit anti-obesity effects. Maeda et al. showed that dietary fucoxanthin (**12**) increases UCP1 expression in the WAT of mice [35]. To the best of our knowledge, this is the first report on carotenoids with the ability to induce UCP1 expression in white adipocytes. Sharma et al. have reported a similar effect of fucoxanthin on 3T3-L1 adipocytes [36]. Besides fucoxanthin, lycopene (**16**) and zeaxanthin (**4**) increased UCP1 expression in 3T3-L1 cells via activation of PPARγ and AMP-activated protein kinase, respectively [37, 38]. Like in brown adipocytes, β-carotene and β-cryptoxanthin (**3**) induced UCP1 expression through RAR activation in white adipocytes [18, 39]. Although the effect of siphonaxanthin (**8**) on the induction of UCP1 has not been evaluated, it has been shown to activate Nrf2 [40], which is essential for white adipocyte browning [41]. Moreover, the Nrf2 activating effect of siphonaxanthin dehydrometabolites (**9**–**11**) that accumulate in the WAT is stronger than that of siphonaxanthin [31]. Therefore, siphonaxanthin and its dehydrometabolites may induce beige adipocyte differentiation via Nrf2 activation. The browning effect of carotenoid metabolites will be an interesting subject for future research.

## Studies in KK-Ay mice

KK-Ay mice were created in Japan in the 1950s by introducing a mutation at the Agouti locus (Ay) into KK mice, which spontaneously develop type 2 diabetes. KK-Ay mice develop severe obesity and hyperglycemia at 7–8 weeks of age and are used as a model of obesity-associated type 2 diabetes for drug development and research on diabetic complications. A Google Scholar search of original papers using the keywords “KK-Ay” and “carotenoids” revealed 12 papers that investigated the anti-obesity and anti-obesity-related disease effects of carotenoids using KK-Ay mice (Table 1). Nine of these were experiments in which fucoxanthin (**12**) was orally ingested, and the remaining included one each of astaxanthin (**6**), bixin (**20**), and siphonaxanthin (**8**). Although the experimental conditions were different, they are briefly compared and discussed in this section.

**Table 1:** Studies in KK-Ay mice

Carotenoid/test sample	No	Ref	Dose	Diet	Period	Sex	Major findings	Remarks
Fucoxanthin-rich (67.4%) oil	**12**	[35]	0.4% in diet	AIN-93G	4 weeks	Female	WAT weight ↓	
							WAT UCP1 ↑	
Fucoxanthin	**12**	[43]	0.1% in diet	AIN-93G	4 weeks	Female	Liver DHA ↑	
			0.2% in diet	AIN-93G	4 weeks	Female	Body weight gain ↓	
							Liver DHA ↑	
Fucoxanthinol	**13**	[43]	0.1% in diet	AIN-93G	4 weeks	Female	Liver ARA ↑	
			0.2% in diet	AIN-93G	4 weeks	Female	Liver ARA ↑	
							Liver DHA ↑	
Fucoxanthin	**12**	[42]	0.1% in diet	AIN-93G	4 weeks	Female	Blood glucose ↓	
							Plasma insulin ↓	
							WAT UCP1 ↔	
							Body weight gain ↔	
							BAT weight gain ↑	
			0.2% in diet	AIN-93G	4 weeks	Female	Body weight gain ↓	
							uWAT weight ↓	
							mWAT weight ↓	
							pWAT and rWAT weight ↓	
							Blood glucose ↓	
							Plasma insulin ↓	
							uWAT UCP1 ↑	
							BAT weight ↑	
Fucoxanthin	**12**	[44]	0.1% in diet	AIN-93G	4 weeks	Female	uWAT weight ↓	
							mWAT weight ↓	
							Liver triacylglycerol ↓	
							Body weight gain ↔	
							BAT weight ↑	
Fucoxanthin	**12**	[45]	0.2% in diet	AIN-93G	4 weeks	Female	Body weight gain ↓	
							WAT weight ↓	
							Blood glucose ↓	
							BAT weight ↑	
Fucoxanthin	**12**	[48]	0.2% in diet	AIN-93G	2 weeks	Female	Blood glucose ↓	
							Serum insulin ↓	
							Skeletal muscle GLUT4 ↑	
							Skeletal muscle insulin signaling ↑	
Fucoxanthin	**12**	[46]	0.2% in diet	AIN-93G	4 weeks	Male	Body weight gain ↓	
							eWAT weight ↓	
							eWAT cholesterol ↓	
							Liver cholesterol ↓	
							Liver LDLR ↓	
							Liver SR-B1 ↓	
							Serum total cholesterol ↑	
							Serum HDL cholesterol ↑	
							Serum non-HDL cholesterol ↑	
							Liver weight ↑	
							Liver PCSK9 mRNA ↑	
Fucoxanthin	**12**	[47]	0.1% in diet	High fat diet	3 weeks	Female	aWAT lipid hydroperoxide ↓	
				(30% fat by weight)			Blood glucose ↓	
							Liver neutral lipid ↓	
							Liver lipid hydroperoxide ↓	
							Body weight gain ↔	
							aWAT weight ↔	
							BAT weight ↑	
Fucoxanthin	**12**	[49]	0.1% in diet	AIN-93G	1 weeks	Female	Liver SCD1 ↓	
			0.1% in diet	AIN-93G	2 weeks	Female	Serum leptin ↓	
							Liver SCD1 ↓	
			0.2% in diet	AIN-93G	2 weeks	Female	Body weight gain ↓	
							vWAT weight ↓	
							Serum leptin ↓	
							Liver SCD1 ↓	
							Liver SCD1 mRNA ↓	
							Liver oleic acid ↓	
							Liver stearic acid ↑	
Astaxanthin	**6**	[50]	10, 50, 100 mg/kg B.W	AIN-76-based diet*	3 weeks	Male	Blood glucose ↓	Gavaged twice a day
							Serum total cholesterol ↓	
							Serum LDL cholesterol ↓	
							Serum triacylglycerol ↓	
							Serum insulin ↓	
							Serum HDL cholesterol ↑	
Bixin	**20**	[51]	0.5% in diet	High fat diet	4 weeks	Male	Serum triacylglycerol ↓	Pair-fed
				(60% fat by calories)			Liver triacylglycerol ↓	
							Body weight gain ↔	
							WAT weight ↔	
							Liver weight ↔	
							Glucose tolerance ↑	
							Serum adiponectin ↑	
							Liver PPARα target genes mRNA expression ↑	
			1.0% in diet	High fat diet	4 weeks	Male	Serum triacylglycerol ↓	
				(60% fat by calories)			Serum glucose ↓	
							Serum insulin ↓	
							Insulin tolerance ↓	
							Liver triacylglycerol ↓	
							Body weight gain ↔	
							WAT weight ↔	
							Liver weight ↔	
							Glucose tolerance ↑	
							Serum adiponectin ↑	
							Liver PPAR target genes mRNA expression ↑	
Siphonaxanthin-rich (73%) fraction	**8**	[26]	1.3 mg/mouse/day	AIN-93G**	6 weeks	Male	mWAT weight ↓	Gavaged once a day
							Body weight gain ↔	
							BAT weight ↔	
							Serum glucose ↔	
							Liver cholesterol ↔	
							Liver triacylglycerol ↔	

↓ decrease; ↑ increase; ↔ no change

^*^Mixture of AIN-76, yolk powder, lard oil, cholesterol (76:13:10:1)

^**^Mice were received 0.1 mL of triolein with or without siphonaxanthin-rich fraction

*No.* compound number, *Ref* reference, *uWAT* uterinary WAT, *mWAT* mesentery WAT, *pWAT* perirenal WAT, *rWAT* retroperitoneal WAT, *eWAT* epididymal WAT, *aWAT* abdominal WAT, *vWAT* visceral WAT, *DHA* docosahexaenoic acid, *ARA* arachidonic acid

KK-Ay mice fed a 0.2% fucoxanthin (**12**)-supplemented AIN-93G diet for 4 weeks showed less weight gain than mice fed a non-supplemented diet, whereas the 0.1% fucoxanthin-supplemented diet had no effect [42, 43]. Hence, there appeared to be a threshold between 0.1 and 0.2%, which is supported by the other studies [44, 45]. In the case of KK-Ay mice, females tend to accumulate fat more easily than males [42], and the above studies were conducted using female KK-Ay mice. Although we could not find any reports comparing sex differences in the effects of fucoxanthin, similar anti-obesity effects have been reported in male KK-Ay mice [46]. As for the weight of WAT, a slight but significant decrease in the weight of uterine and mesenteric WAT was reported after 4 weeks of feeding with a 0.1% fucoxanthin-supplemented diet [44]. However, under the same experimental conditions, another study showed no change in the WAT weights [42], suggesting that the decreasing effect of a 0.1% fucoxanthin-supplemented diet on WAT weight is weak. In these experiments, the weight of brown adipose tissue (BAT) increased after 4 weeks of feeding on a 0.1% fucoxanthin-supplemented diet [42, 44]. In addition, KK-Ay mice fed a high-fat diet containing 30% fat and 0.1% fucoxanthin for 3 weeks exhibited an increase in BAT weight without a decrease in abdominal WAT weight [47], indicating that fucoxanthin has a stronger effect on BAT than on WAT. UCP1 expression in WAT was also observed in KK-Ay mice fed a 0.2% fucoxanthin-supplemented diet, whereas the 0.1% fucoxanthin-supplemented diet had no effect [35, 42].

The effects of fucoxanthin (**12**) on tissues other than the adipose tissue have also been investigated in KK-Ay mice. Decreases in blood glucose and plasma insulin levels have been reported after 4 weeks of feeding on 0.1 and 0.2% fucoxanthin-supplemented diets [42, 45, 47, 48]. The skeletal muscle responds to insulin by translocating glucose transporter type 4 (GLUT4) to the plasma membrane and taking up glucose, thereby lowering blood glucose levels. A 0.2% fucoxanthin-supplemented diet enhanced insulin signaling in the skeletal muscle of KK-Ay mice and increased GLUT4 expression and translocation to the plasma membrane [48].

In addition, a unique phenomenon has been reported in the liver, in which 4 weeks of a diet supplemented with fucoxanthin (**12**) or its deacetylated form, fucoxanthinol (**13**), increased docosahexaenoic acid and arachidonic acid levels [43]. Moreover, only 1 week of a 0.1% fucoxanthin-supplemented diet significantly decreased the expression of stearoyl-CoA desaturase 1 (SCD1), the rate-limiting enzyme in the monounsaturated fatty acid synthesis pathway [49]. Because no similar SCD1 downregulation was observed in Ob/Ob mice, which lack leptin, a hormone produced by the adipose tissue, fucoxanthin is believed to alter liver SCD1 expression by affecting leptin signaling [49].

Fucoxanthin (**12**) affects not only the fatty acid composition of the liver but also cholesterol; feeding a 0.2% fucoxanthin-supplemented diet for 4 weeks lowered the amount of cholesterol in the liver [46]. In fucoxanthin-fed KK-Ay mice, the protein expression levels of low-density lipoprotein (LDL) receptor, which takes up LDL cholesterol from the blood, decreased, whereas the mRNA expression levels of proprotein convertase subtilisin/kexin type 9 (PCSK9), which promotes LDL receptor degradation, increased in the liver. The expression of scavenger receptor class B member 1 (SR-B1), which is responsible for the uptake of high-density lipoprotein (HDL) cholesterol by the liver, also decreased. Furthermore, serum total cholesterol, non-HDL cholesterol, and HDL cholesterol concentrations were increased, suggesting that fucoxanthin reduced cholesterol accumulation in the liver by inhibiting cholesterol uptake from the blood. The simultaneous increase in cholesterol, generally referred to as “bad” and “good” cholesterol, would require careful interpretation of the health implications of this activity.

Although the experimental conditions were very different, except for the use of KK-Ay mice (Table 1), astaxanthin (**6**) has also been reported to increase serum HDL cholesterol concentration [50]. In this study, astaxanthin administration significantly decreased serum LDL cholesterol and total cholesterol concentrations, as well as blood glucose and serum insulin levels. These results raise interest in the relationship between carotenoids and serum cholesterol levels.

Bixin (**20**) has an agonist effect on PPARγ [21] but also activates PPARα. Using a reporter gene assay, Goto et al. showed that bixin activates PPARα and that feeding a high-fat diet (60% fat by calories) supplemented with 0.5 or 1.0% bixin for 4 weeks reduced liver triacylglycerol levels in KK-Ay mice [51]. They also reported increased expression of PPARα-regulated and fatty acid β-oxidation-related genes. The 1.0% bixin-supplemented diet also reduced blood glucose and serum insulin levels, and improved glucose tolerance and insulin resistance, indicating that bixin has an ameliorative effect on triacylglycerol accumulation in the liver through activation of PPARα and on obesity-induced abnormalities of carbohydrate metabolism.

We previously fed KK-Ay mice with 1.3 mg siphonaxanthin (**8**) (purity = 73%) per day for 6 weeks and showed that siphonaxanthin administration significantly decreased mesenteric WAT weight [26]. The food intake of the KK-Ay mice in this study was approximately 4.3 g per day. This means that they ingested 0.03% siphonaxanthin relative to their total food intake. Hence, siphonaxanthin could exhibit a decreasing effect on WAT weight at a lower dose than fucoxanthin (**12**), astaxanthin (**6**), and bixin (**20**). In contrast, there was no increase in BAT weight, no decrease in liver cholesterol and triacylglycerol levels, or no decrease in blood glucose levels, as reported in KK-Ay mouse studies with other carotenoids. Tissue distribution analyses of ingested siphonaxanthin in KK-Ay and also ICR mice showed that siphonaxanthin accumulated best in mesenteric WAT, except in the digestive tract [26, 30]. Although the mechanism by which siphonaxanthin accumulates in mesenteric WAT is unclear, it has been observed that the biological activity of siphonaxanthin is in tissues with high accumulation and not in tissues with low accumulation, including BAT, liver, and skeletal muscle. The tissue distribution of ingested siphonaxanthin and the mechanism of its anti-obesity effects may be different from those of other carotenoids; thus, a combined effect could be expected.

## Studies in ob/ob mice

The Ob/Ob mouse was discovered in 1950 at Jackson Laboratory in the United States. They lack the hormone leptin, which is secreted by adipocytes and has the function of suppressing feeding and increasing energy metabolism. Hence, they become obese owing to overeating. This is a well-studied model of obesity and type 2 diabetes mellitus. A search for original papers using Google Scholar with the keywords “Ob/Ob” and “carotenoids” found only five, many of which were newer than the reports of carotenoid studies using KK-Ay mice (Table 2). The carotenoids evaluated were apo-10′-lycopenoic acid (**18**), a metabolite of lycopene (**16**), astaxanthin (**6**), fucoxanthin (**12**) (two studies), and siphonaxanthin (**8**), and the studies were conducted over longer periods, from 6 to 40 weeks. In addition, most studies have focused on the liver rather than the adipose tissue. In this section, we discuss the effects of siphonaxanthin on the obesity-related liver abnormalities.

**Table 2:** Studies in Ob/Ob mice

Carotenoid/test sample	No	Ref	Dose	Diet	Period	Sex	Major findings	Remarks
Apo-10′-lycopenoic acid	**18**	[53]	0.004% in diet	Lieber-DeCarli	16 weeks	Male	Liver steatosis ↓	Pair-fed
				high-fat liquid diet*			Liver acetylated FOXO1 ↓	
							Liver ACACA mRNA ↓	
							Liver triacylglycerol ↔	
							Liver SIRT1 ↑	
							Liver SITR1 mRNA ↑	
Astaxanthin	**6**	[54]	0.02% in diet	High fat diet	10 weeks	Male	Liver weight ↓	
				(60% fat by calories)			Liver steatosis ↓	
							Liver triacylglycerol ↓	
							Body weight gain ↔	
							eWAT weight ↔	
Astaxanthin	**6**	[54]	0.02% in diet	Atherogenic	12 weeks	Male	Liver triacylglycerol ↓	
				high-fat diet**			Liver cholesterol ↓	
							Liver fibrosis ↓	
							Body weight gain ↔	
							Liver weight ↔	
Fucoxanthin	**12**	[49]	0.2% in diet	not specified	4 weeks	Male	Body weight gain ↔	
							Blood glucose ↔	
							Liver SCD1 ↔	
							Liver oleic acid ↔	
							Liver stearic acid ↔	
Fucoxanthin	**12**	[55]	0.2% in diet	not specified	24 weeks	Male	Liver steatosis ↓	
							Liver triacylglycerol ↓	
							Liver Nrf1 ↑	
							Liver TFAM ↑	
Fucoxanthin	**12**	[56]	0.05% in diet	not specified	40 weeks	Male	Blood glucose ↓	
							Insulin tolerance ↓	
							Body weight gain ↔	
							Glucose tolerance ↑	
							Skeletal muscle TFAM mRNA ↑	
Siphonaxanthin-rich (68%) fraction	**8**	[40]	0.016% in dier	High fat diet***	6 weeks	Male	Plasma alanine transaminase activity ↓	
							Liver oxidative stress ↓	
							Body weight gain ↔	
							mWAT weight ↔	
							Liver weight ↔	
							Liver steatosis ↔	
							Liver triacylglycerol ↔	
							Liver cholesterol ↔	

↓ decrease; ↑ increase; ↔ no change

^*^High fat diet (60% fat, 22% carbohydrate, and 18% protein by calories)

^**^High fat diet (60% fat by calories, and 1.25% cholesterol and 0.5% sodium cholate by weight)

^***^High fat diet (45% fat by calories)

*No.* compound number, *Ref* reference, *eWAT* epididymal WAT, *mWAT* mesentery WAT

Apo-10′-lycopenoic acid (**18**) is an apocarotenoid formed from lycopene (**16**) by β,β-carotene-9′,10′-oxygenase 2 (BCO2) and NAD^+^-dependent dehydrogenase [52]. Biological activities of lycopene in vivo may be mediated in part by apo-10′-lycopenoic acid. Jayong et al. reported that Ob/Ob mice fed a Lieber-DeCarli high-fat liquid diet (60% fat by calories) containing 0.004% apo-10′-lycopenoic acid for 16 weeks exhibited lower hepatic steatosis [53]. The high expression levels and activity of sirtuin 1 (SIRT1), a regulator of lipid metabolism, in the liver are considered a mechanism of action.

There has been one detailed report on the effects of astaxanthin (**6**) on the livers of Ob/Ob mice. Ob/Ob mice fed a high-fat diet (60% fat by calories) supplemented with 0.02% astaxanthin for 10 weeks showed significant reductions in liver weight and triacylglycerol content [54]. The same research group examined the effects of astaxanthin on MASH in Ob/Ob mice fed an atherogenic high-fat diet (60% fat by calories) containing cholesterol and cholic acid. Interestingly, compared to α-tocopherol, which is used to treat human MASH, astaxanthin showed comparable ameliorative effects (such as the decrease in liver triacylglycerol and cholesterol levels) [54]. Similar ameliorative effects were observed when an astaxanthin-supplemented diet was administered after the onset of MASH. Furthermore, the authors reported that astaxanthin ingestion ameliorated fatty liver in patients with MASH. Although further interventional studies in humans are required, astaxanthin may have ameliorative effects on MAFLD and MASH.

It was recently reported that 24 weeks of diet supplementation with 0.2% fucoxanthin (**12**) improved hepatic steatosis and decreased liver triacylglycerol levels in Ob/Ob mice [55]. In addition, mice fed a 0.05% fucoxanthin-supplemented diet for 40 weeks showed decreased blood glucose and insulin levels, and improved glucose tolerance and insulin sensitivity [56]. Increased expression of genes involved in mitochondrial biosynthesis in the liver [55] and skeletal muscle [56] is considered to be the molecular mechanism by which fucoxanthin improves hepatic steatosis and diabetes mellitus. Considering that fucoxanthin increased mitochondrial UCP1 expression in WAT and the weight of BAT, which is enriched in mitochondria, it is likely that fucoxanthin exerts its anti-obesity effects by acting on the mitochondria.

The ingestion of 0.016% siphonaxanthin (**8**) (purity = 68%)-supplemented high-fat diet (45% fat by calories) for 6 weeks did not decrease mesenteric WAT weight, liver triacylglycerol and cholesterol levels, or hepatic steatosis compared to control mice fed a high-fat diet without supplementation [40]. However, it significantly reduced plasma alanine transaminase activity and liver thiobarbituric acid-reactive substances levels. These results suggest that siphonaxanthin ingestion improved hepatic oxidative stress, which is involved in the pathogenesis of MAFLD and MASH. We previously showed that siphonaxanthin suppressed liver X receptor activation and triacylglycerol accumulation in human liver-derived HepG2 cells more strongly than astaxanthin (**6**) and fucoxanthin (**12**) [57]. Taken together, these results suggest that siphonaxanthin has the potential to ameliorate the symptoms of MAFLD and obesity. Future experiments using higher doses or long-term feeding are required.

## Future perspectives

Comparative studies at the cellular level have shown that siphonaxanthin (**8**) has stronger biological activity than other carotenoids [5, 6, 26, 57–59]. In addition, siphonaxanthin is characterized by its tendency to accumulate in mesenteric WAT [26, 30] and be metabolized into highly active dehydrometabolites [31]. Unfortunately, no clear anti-obesity effects have been observed in animal studies, most likely because of the low doses [26, 40]. Astaxanthin (**6**) and fucoxanthin (**12**) are produced in large quantities by microalgae such as *Haematococcus gracilis* and *Phaeodactylum tricornutum*. If microalgae could be utilized to mass produce siphonaxanthin, it would be possible to conduct animal experiments using higher doses or long-term feeding. Thus, the anti-obesity effects of siphonaxanthin were expected to be clearer.
